# Unconditioned Stimulus Revaluation to Promote Conditioned Fear Extinction in the Memory Reconsolidation Window

**DOI:** 10.1371/journal.pone.0101589

**Published:** 2014-07-17

**Authors:** Xiang-Xing Zeng, Juan Du, Chu-Qun Zhuang, Jun-Hua Zhang, Yan-Lei Jia, Xi-Fu Zheng

**Affiliations:** 1 Center for Studies of Psychological Application, South China Normal University, Guangzhou, China; 2 Health Service Centers in Communities, South China Normal University, Guangzhou, China; Radboud University, Netherlands

## Abstract

The retrieval-extinction paradigm, which disrupts the reconsolidation of fear memories in humans, is a non-invasive technique that can be used to prevent the return of fear in humans. In the present study, unconditioned stimulus revaluation was applied in the retrieval-extinction paradigm to investigate its promotion of conditioned fear extinction in the memory reconsolidation window after participants acquired conditioned fear. This experiment comprised three stages (acquisition, unconditioned stimulus revaluation, retrieval-extinction) and three methods for indexing fear (unconditioned stimulus expectancy, skin conductance response, conditioned stimulus pleasure rating). After the acquisition phase, we decreased the intensity of the unconditioned stimulus in one group (devaluation) and maintained constant for the other group (control). The results indicated that both groups exhibited similar levels of unconditioned stimulus expectancy, but the devaluation group had significantly smaller skin conductance responses and exhibited a growth in conditioned stimulus + pleasure. Thus, our findings indicate unconditioned stimulus revaluation effectively promoted the extinction of conditioned fear within the memory reconsolidation window.

## Introduction

The extinction of conditioned fear has been extensively examined because these negative emotions have the potential to influence normal life in both humans and animals. Conditioned fear refers to the phenomenon wherein a neutral stimulus (conditioned stimulus, CS) that does not initially induce fear in an individual begins to do so after it is repeatedly paired with an intrinsically aversive consequence (unconditioned stimulus, US). The repeated pairing of the CS with the US is believed to lead to an association between CS and US (CS-US) that enables the CS to elicit conditioned fear (conditioned response, CR) [1]–[6]. However, if the CS is repeatedly presented without the US (CS/no-US), fear of the CS will gradually extinguish [7]–[10]. Extinction-like exposure therapy appeared to be an effective method of treating conditioned fear [11], but the fear tended to recur easily in many situations [12]–[13]. In addition, pharmacological manipulations are considered effective, but have a number of negative side effects in humans [14]–[15].

Recently, a new retrieval-extinction (Ret-Ext) technique based on memory reconsolidation theory has been proposed as a means of disrupting previous fear memory reconsolidation and providing long-lasting prevention against conditioned fear relapse [15]. Memory reconsolidation theory states that the memory consolidation process need to repeat many times. Specifically, consolidated memories transiently return to a labile state upon each subsequent retrieval and must be reconsolidated. The memory reconsolidation process persists for about 6 hours, during which plasticity changes occur; during this time, the memories are vulnerable to interference and more likely to be rewritten and erased [16]–[18]. In the Ret-Ext technique, using a retrieval trial activates a consolidated fear memory, and subsequently extinction training is presented during the reconsolidation time window; as a result, the extinction training might decrease the valence of the fear stimulus and consequently rewrite or erase the conditioned fear memory. Through a series of animal experiments, it was shown that the Ret-Ext technique effectively prevented the effect of spontaneous recovery, reinstatement and fear reacquisition ability. Subsequent trials of the technique in human subjects indicated a reduction in fear responses that lasted a least a year [19]. However, some results suggest that the Ret-Ext technique did not effectively extinguish the conditioned fear [15], [20]–[23]. One of the primary concerns is whether the retrieval trial effectively activates the consolidated memory and opens the memory consolidation window. At present, there are two main methods of memory activation used in reconsolidation studies: One is the presentation of an isolated retrieval trial CS and the other is the presentation of the CS paired with the US [24]. Recently, a new unconditioned stimulus revaluation (US-revaluation) paradigm has been used to explore the action mechanism of US during the process of conditioned fear acquisition [25]. This paradigm states that after acquisition, presenting a US with a modified valence can affect the fear response during the test phase. For example, the post-acquisition presentation of a US of decreased intensity in a subsequent test session results in the weakening of CS fear response. These finding suggest the crux of Pavlovian conditioning is the association between the CS and the presentation of the US, and that the current value of US presentation is an important determinant of whether a CR is elicited by CS. In addition, another some studies find that US-revaluation not only leads to corresponding fear response changes for the pre-associated CS, but also reactivates the original fear memory [26].

So far, the focus of studies on US-revaluation has been the test session [25], [27]–[28]. But the application of US-revaluation towards the extinction of conditioned fear has not been explored. Given that US-revaluation can affect the conditioned fear response and reactivate the original memory, in the present experiment, we combine the US-revaluation paradigm and the Ret-Ext technique to explore whether US-revaluation within the Ext-Ret technique can promote the extinction of conditioned fear. Based on previous studies, we divided participants into two groups: at one day post-acquisition, the intensity of the US was decreased for one group (devaluation) and held constant for the other group (control), and 15 min after US-revaluation (within the memory reconsolidation window), extinction training began. Three methods for indexing fear [unconditioned stimulus expectancy (US-expectancy), skin conductance response (SCR), conditioned stimulus + (CS+) pleasure rating] were used. We hypothesized that the fear response of the devaluation group would be significantly lower than that of the control group during the Ret-Ext technique, and the CS+ pleasure of the devaluation group would be significantly greater than that of the control group if the US-revaluation changed the conditioned fear response.

## Materials and Methods

### Ethics statement

The experimental procedure was approved by the ethics committee of South China Normal University. All participants provided written informed consent before taking part in the experiments.

### Participants

Thirty-five undergraduate students (11 men, 24 women) from South China Normal University were participants in this study. Participants were right-handed, had normal or corrected-to-normal vision, and had not been diagnosed with any somatic diseases or psychological disorders. Participants were randomly assigned to one of two conditions with the restriction that conditions were matched on State-Trait Anxiety Inventory scores [29] (State: *M*_devaluation_ = 37.43, *SD* = 8.30, *M*_control_ = 34.75, *SD* = 6.37, *t* = 0.99 *p*>0.05; Trait: *M*_devaluation_ = 42.93, *SD* = 9.03, *M*_control_ = 40.44, *SD* = 7.69, *t* = 0.82 *p*>0.05). Four participants were excluded from the final analysis due to technical problems and voluntary withdrawal, leaving a final sample of 31 participants [devaluation group: *n* = 16 (6 men, 10 women); control group: *n* = 15 (5 men, 10 women)] between the ages of 18 and 22 years (*M* = 19.8, *SD = *1.70). Participants were modestly compensated for their participation in the experiment.

### Apparatus and Materials

#### Stimuli

A negative affective sound (CASS numbers 127, arousal: *M* = 2.04, *SD* = 0.89; valence: *M* = 7.06, *SD* = 1.08) that served as the US was selected from the Chinese Affective Sound System (CASS) [30]–[31]. For the CS, we employed images of two different geometries (triangle/square): one geometry (CS+) was paired with an affective sound, whereas the other geometry was not paired with sound (CS−). Assignment of the slides as CS+ and CS− was counterbalanced across participants. The images used in this experiment were identical in size and resolution.

#### US-expectancy measure

Following CS presentation, participants rated their expectation of the US. The question “Is there a negative affective sound?” was presented using a 10-point scale, from 0 (*certainly no negative affective sound*) to 9 (*certainly a negative affective sound*). Participants rated their expectancy of a negative affective sound by pressing the corresponding number key.

#### Skin conductance response

SCR was measured using the Spirit NeXus-10 Bio Trace system. Two Ag/AgCI electrodes were attached to the tips of the second and third fingers of the participant’s non-preferred hand. The electrodes were connected to the GSR100 c module, which recorded SCR at 120 Hz. SCR elicited by the CS were determined by calculating the difference between the baseline average (i.e., 5 s before CS onset) and peak response within the 1–8 s window following stimulus onset. A minimum response criterion of 0.02 micro Siemens (µS) was used. All other responses were scored as zero and remained in the analyses [14], [32]–[33]. The raw SCR scores were square root-transformed to normalize the distribution.

#### CS pleasure ratings

All participants were required to rate the valance of the CS+ after acquisition, revaluation, and extinction on a 9-point scale from 1 (*no pleasure*) to 9 (*very much pleasure*). This scale is designed to assess the degree of fear participants elicited by CS+ [34].

### Experimental procedure

The experiment consisted of several phases and was conducted over the course of two days (separated by 24 h). Participants sat behind a table with a 21-inch LCD monitor at a distance of 50 cm in a sound-attenuated and air-conditioned room (25°C). The monitor ran at a refresh rate of 60 Hz and had a resolution of 1024×768 pixels. The software package E-Prime 1.0 was used for stimuli presentation and data collection.

#### Day 1: Acquisition

Prior to the experiment, all study procedures were explained in detail to participants and any questions were answered. We asked participants questions about their general health and possible medical conditions to determine participation eligibility. If a participant was eligible, written informed consent was obtained and the State-Trait Anxiety Inventory (STAI) was administered.

After attachment of the SCR monitors, participants were informed that one of the slides (CS+) would be followed by a negative affective sound (US, 100-DB), whereas the other slides (CS–) would be followed by a black screen. Participants were told their task was to learn to predict the occurrence of the US. Participants were required to rate their expectancy to the US after CS presentation by pressing the corresponding number key with the preferred hand on a 10-point rating scale when the slide was presented (rate expectancy; RE) before US presentation.

In the acquisition phase, the CS− and CS+ were presented 8 times for 8 s. Following CS presentation, the US or the black screen was shown for 8 s. The RE was presented between CS and US and would disappear when subjects pressed a key. Trial type order was randomized within blocks (i.e., CS–, CS+). Intertrial intervals were 800 ms. The acquisition phase consisted of 8 CS+ and 8 CS− presentations. Prior to the formal experiments, participants completed a practice test to ensure they adequately understood the experimental procedure.

All participants were asked to rate CS+ pleasure after acquisition. At the conclusion of the experiment, participants were explicitly instructed to remember what they had learned during acquisition. These instructions were provided to enhance participants’ retention of the CS-US association in the following experiment and to prevent participants from erroneously expecting a different association scheme during the subsequent day.

#### Day 2: Memory retrieval and revaluation vs

**extinction.** A 24 h break after acquisition was inserted to ensure memory consolidation.

On Day 2, SCR electrodes were attached, and all participants received three presentations of the US in the absence of either CS. The background of the visual display was blue during the revaluation phase [25]. The US revaluation phase followed the memory reactivation phase. For the control group, the US intensity during the revaluation phase was the same as it had been in the acquisition phase. For the devaluation group, in accordance with previous studies, the US intensity during the revaluation phase was modified from the 100-DB tone used during the acquisition phase to a 60-DB tone [35]. A resting period of 15 min was inserted and all participants were asked to rate the CS+ pleasure after US-revaluation [14], [19]. During this break, participants conversed with an experiment assistant. Then, participants were told that the same two slides (CS) would be presented and were asked to remember what they had learned during acquisition, but the two sliders (CS+, CS−) were followed by a black screen. Further instructions regarding the expectancy ratings were similar to Day 1. The extinction phase consisted of 12 CS− and 12 CS+ presentations. All participants were asked to rate the CS+ pleasure after extinction.

### Statistical analysis

All analyses were performed with SPSS 19.0. CS+ pleasure, SCR, and US-expectancy ratings were analyzed using a mixed analysis of variance (ANOVA) for repeated measures with the groups as the between-subjects factor, and stimulus (CS− vs. CS+) and trial (i.e., stimulus presentation) as within-subjects factors. Planned comparisons were performed for each condition separately. Missing data points were excluded from the analyses. Significance level was set at *p*<0.05.

## Results

### US-expectancy

**Figure 1:**
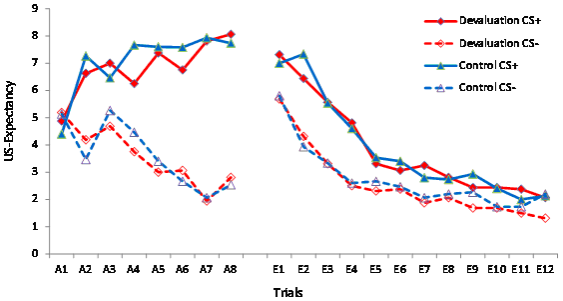
Mean expectancy scores. The US-expectancy of the CS+ and CS− trials during acquisition (A1–A8) and retrieval-extinction (E1–E12) for the devaluation and control groups.

The mean expectancy score of US for each CS+ and CS−trial presentation in two groups was shown below (see Figure 1).

A trial × CS type × group repeated measures ANOVA of US-expectancy ratings revealed no differences between the two conditions [*F*(1, 29) = 1.06, *p*>0.05, Partial *η* ^2^ = 0.03]. The main effects tests identified a significant difference between CS types [*F*(1, 29) = 85.49, *p*<0.01, Partial *η* ^2^ = 0.74]. The main effects for trials revealed a significant difference, [*F*(7, 53) = 3.39, *p*<0.01, Partial *η* ^2^ = 0.11]. There was a significant interaction between CS type and trial [*F*(7, 53) = 26.72, *p*<0.01, Partial *η* ^2^ = 0.48] characterized by a higher expectancy rating for the CS+ than for the CS− trials. The data indicated that participants learned to expect the US on CS+ trials and not to expect the US on CS− trails during acquisition, and that all participants acquired conditioned fear at the same level.

The US-expectancy of the CS+ remained stable from the last acquisition trial (trial A8) to the first extinction trial (trial E1; 24 h later; after US-revaluation) in the two conditions (Devaluation: *M*_A8_ = 8.06, *SD* = 1.06; *M*_E1_ = 7.31, *SD* = 1.85, *t* = 2.08, *p*>0.05; Control: *M*_A8_ = 7.73, *SD* = 1.38; *M*_E1_ = 7.00, *SD* = 2.29, *t* = 1.46, *p*>0.05). This result shows that US presentation effectively reactivated the memory of the consolidated fear during the Ret-Ext procedure on Day 2.

For the extinction phase, the main effects tests identified a significant difference for CS type and trial [CS types: *F*(1, 29) = 19.40, *p*<0.01, Partial *η* ^2^ = 0.40; trial: *F*(11, 319) = 60.07, *p*<0.01, Partial *η* ^2^ = 0.67]. There was a significant interaction between CS type and trial [*F*(11, 319) = 6.02, *p*<0.01, Partial *η* ^2^ = 0.17]. There were no significant differences between the two conditions [*F*(1, 29) = 0.06, *p*>0.05, Partial *η* ^2^ = 0.02]. The data indicated that the conditioned fear was effectively extinguished in the two groups. And that the devaluation and control groups showed similar levels of US-expectancy during extinction after US-revaluation.

### Skin conductance response

**Figure 2:**
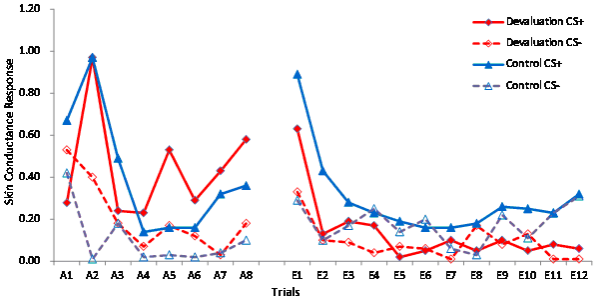
Mean skin conductance response. The SCR of the CS+ and CS− trials during acquisition (A1–A8) and retrieval-extinction (E1–E12) for the devaluation and control groups.

The mean score of SCR for each CS+ and CS−trial presentation in two groups was shown below (see Figure 2).

A trial × group × CS type repeated measures ANOVA indicated that, during the acquisition phase, participants began to exhibit a larger SCR to CS+ trials than to CS−trials; the difference between the SCR elicited by the CS+ and CS−was significant [F(1, 29) = 9.20, p<0.05, Partial η2 = 0.24]. A CS type × group ANOVA did not detect any differences between the devaluation and control groups [F(1, 29) = 0.37, p>0.05, Partial η2 = 0.17]. The data suggested both groups successfully learned conditioned fear on Day 1.

The SCR of the CS+ remained stable from the last acquisition trial (trial A8) to the first extinction trial (trial E1; 24 h later; after US-revaluation) in the devaluation group (*M*_A8_ = 0.72, *SD* = 0.81; *M*_E1_ = 0.69, *SD* = 0.42, *t* = 0.13, *p*>0.05), and the SCR of the CS+ of the first extinction trial (trial E1; 24 h later; after US-revaluation) was significantly larger than the SCR of the last acquisition trial (trial A8) in the control group (*M*_A8_ = 0.38, *SD* = 0.61; *M*_E1_ = 0.89, *SD* = 0.49, *t* = −2.18, *p*<0.05). These SCR data showed that US presentation effectively reactivated the fear response during the retrieval activation process on Day 2.

A trial × CS type repeated measures ANOVA comparing the devaluation and control groups during the retrieval-extinction phase indicated the following. In the devaluation group, the main effect for trials was significant [*F*(11, 165) = 4.88, *p*<0.01, Partial *η* ^2^ = 0.25], but the main effect for CS type was not significant [*F*(1, 15) = 4.05, *p*>0.05, Partial *η* ^2^ = 0.62]. In the control group, neither the main effect for trials [*F*(11, 154) = 1.51, *p*>0.01, Partial *η* ^2^ = 0.09] nor the main effect for CS type [*F*(1, 14) = 2.69, *p*>0.05, Partial *η* ^2^ = 0.16] was significant. A trial × group repeated measures ANOVA yielded a significant main effect for group [*F*(11, 319) = 0.81, *p*>0.05, Partial *η* ^2^ = 0.03]. The SCR of the CS+ was significantly larger in the control group than in the devaluation group [*F*(1, 29) = 6.12, *p*<0.05, Partial *η* ^2^ = 0.17]. These data indicate both groups successfully learned the fear extinction, and that extinction learning was more pronounced in the devaluation group relative to the control group.

### CS+ valance ratings

**Figure 3:**
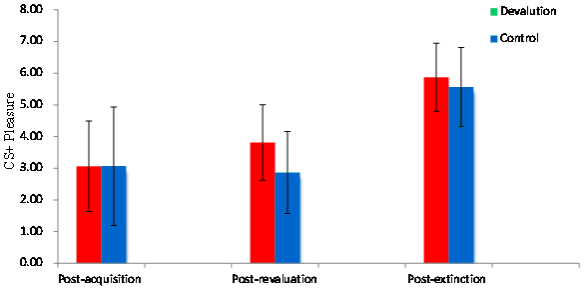
CS+ pleasure ratings. Post-acquisition, post-revaluation, and post-extinction for the devaluation and control groups.

A time × group repeated measures ANOVA indicated the mean CS+ pleasure rating significantly increased from post-acquisition to post-extinction between the two groups [*F*(2, 29) = 18, 57, *p*<0.05, Partial *η* ^2^ = 0.65](see Figure 3). An independent samples *t*-test for CS+ was conducted to assess differences between the devaluation and control groups. In post-acquisition, a significant difference was not observed between the mean CS+ pleasure ratings of the devaluation and control groups (*M_d_*_evaluation_ = 3.06, *SD* = 1.43; *M*_control_ = 3.06, *SD* = 1.87, *t* = 0.005, *p*>0.05). In post-revaluation, the mean CS+ pleasure ratings of the devaluation group were slightly greater than that of the control group, and the difference reached significance (*M*_devaluation_ = 3.81, *SD* = 1.19; *M*_control_ = 2.87, *SD* = 1.29, *t* = 1.17, *p*<0.05). In post-extinction, the mean CS+ pleasure ratings of the devaluation group training was moderately greater than that of the control group, but this difference was not significant (*M*_devaluation_ = 5.87, *SD* = 1.07; *M*_control_ = 5.56, *SD* = 1.25, *t* = 0.39, *p*>0.05). Taken together, these data indicated the CS+ pleasure of the devaluation group was significantly greater than that of the control group, suggesting US-devaluation lead to a greater extinction effect (see Figure 3).

## Discussion

In the present study, US-devaluation was combined with the Ret-Ext paradigm, and results indicated that US-devaluation promoted SCR reduction, but did not influence US-expectancy. In the experiment, the mean CS+ pleasure rating significantly increased from post-acquisition to post-extinction between the two groups, which indicated the conditioned fear was successfully extinguished in both groups, but that US-devaluation leaded to a greater extinction effect. These results suggested US-revaluation activated the reconsolidation of the fear memory upon retrieval and leaded to a progressive deconsolidation of the memory followed by the assignment of a new valence to the CS during the extinction phase; this change in valence ultimately resulted in the dissociation between US-expectancy and SCR [21]. The US-revaluation effects observed in the reconsolidation time window are consistent with a number of previous findings [33]–[36].

In the present study, there was no difference in the US-expectancy between the devaluation and control groups. However, the SCR of the devaluation group was significantly lower than that of the controls, which indicated a revaluation effect on SCR, but not US-expectancy. Thus, there was a dissociation between measures, which persisted through the extinction phase. The dissociation effects observed in the present study are consistent with other some experiments [25], [27], [36]–[37], supporting the idea that there are different types of memory formed during the same training procedure. The dual process theory states that implicit performance and explicit performance are dissociable from one another [25], [38]. Explicit performance and implicit performance correspond to two different memory systems: The former involves the declarative memory of the learned fear association between the CS and US; the latter involves procedural memory for the acquisition and expression of a fear response. These two different memory systems depend on different brain regions: Declarative memory depends on the hippocampal complex; while procedural memory is mediated by the amygdala [3], [39]–[40]. Studies of brain-injury patients also indicate the two memory systems are dissociation: Patients with amygdala damage can establish an association between the CS and US, but do not express physiological fear responses; by contrast, patients with hippocampal damage express the fear response, but do not associate the CS and US [41]–[42]. Even though the amygdala and hippocampal complex can operate independently, they also interact in a subtle but important way [20], [41], [43]: The hippocampal dependent declarative memories can lead to activation of the amygdala, which mediates the associated emotional reaction. This is the reason why relapse to conditioned fear occurs easily. As applied to the present case, US-expectancy is the explicit perception of association between the CS and US and is mediated by cognition, while the SCR is an implicit biological response. Hence, we observed double dissociation of the US-expectancy and SCR of conditioned fear. The SCR of the devaluation group was significantly lower than that of the controls, and US presentation activates the reconsolidation of the fear memory. However, because the intensity of the US was decreased, the fear valence of the CS was updated and the reconsolidation of the fear memory was disrupted. The revaluation mechanism does not depend on rewriting the declarative memory for the learned fear association between CS and US; rather, it depends on rewriting the procedural memory of the fear response [25], [36]. Thus, the fear responses are significantly reduced, which is consistent with previous findings [15], [19].

During the acquisition phase, the valence of US fear was delivered to CS+, thus resulting in participants fearing the CS+. After acquisition of conditioned fear, the degree of perceived CS+ pleasure did not differ between the devaluation and control groups, suggesting the two groups had similar levels of CS+ delivered by US fear. However, after revaluation, CS+ pleasure was slightly greater in the devaluation group than in the control group, which may have resulted from the incorporation of the new CS+ fear valence into the restructured memory [25]. When presented with a US of decreased intensity, participants reconstructed their cognition regarding CS+ fear valence and revised the fear memory during reconsolidation. Then, the fear response was further decreased through extinction training after the US-revaluation. The current results suggest the US intensity change may have a more important role during retrieval-extinction. However, the effects on renewal, spontaneous recovery, and reinstatement were not examined. Further study is required to determine whether the presentation of the CS or US as a retrieval trial would be most effective during retrieval-extinction. In conclusion, US-revaluation does not change the old association between the CS and US, but does change the fear valence. Since US-revaluation changes the value of the US without affecting the CS-US contingency, the memory reconsolidation of the old conditioned fear is interrupted when US-revaluation is applied within the memory reconsolidation window, which consequently promotes the extinction of the fear response. Thus, US-revaluation may represent a non-invasive technique that can be used to promote safely the extinction of conditioned fear in humans.

## Supporting Information

File S1 **Data S1–S3.** (Excel).
(RAR)
